# Bioavailability of Ferric Pyrophosphate and Ferric Orthophosphate with/without Extrusion and/or Citric Acid and/or Trisodium Citrate in Rats and In Vitro INFOGEST Digestion and Caco-2 Cell Model

**DOI:** 10.1016/j.cdnut.2026.107718

**Published:** 2026-05-18

**Authors:** Jiejia Zhang, David B Akinbo, Erin J Ward, Hafiz AR Suleria, Tucker Graff, Michael Joseph, Mehreen Iftikhar, Sajid Alavi, Brian L Lindshield

**Affiliations:** 1 School of Health Sciences, College of Human Health and Sciences, Kansas State University, Manhattan, KS, United States; 2 Department of Grain and Food Science, College of Agriculture, Kansas State University, Manhattan, KS, United States

**Keywords:** iron bioavailability, ferric pyrophosphate, ferric orthophosphate, extrusion, citric acid, trisodium citrate, rice

## Abstract

**Background:**

Iron absorption of fortified rice and/or extruded rice flour could be significantly increased with the addition of ferric pyrophosphate (FePP)/micronized FePP (μFePP)/ferric orthophosphate (FePO_4_), citric acid (CA), and trisodium citrate (TSC) at low (1:0.1:2.1) and high (1:0.3:5.5) molar ratios. Thus, 2 rat studies and a Caco-2 cell study were carried out to examine the iron bioavailability of different formulations and food processing strategies.

**Objectives:**

To determine the iron bioavailability of rice and extruded rice flour fortified with the different formulations of FePP/μFePP/FePO_4_, CA, and TSC in rats and the INFOGEST digestion/Caco-2 cell model.

**Methods:**

In rat studies, Sprague-Dawley rats were randomly assigned iron-fortified rice diets using the prophylactic-preventative method. The iron bioavailability was determined by measuring the concentrations of hematological parameters and hepatic iron. In the cell-culture study, iron-fortified extruded rice flour was digested with the INFOGEST digestion method. The iron solubility was analyzed with inductively coupled plasma–optical emission spectrometry. The iron bioavailability was determined by measuring ferritin concentrations.

**Results:**

In rat study 1, hemoglobin and hepatic iron levels were significantly higher in the FePP and μFePP groups compared with the FePO_4_ and control groups. In rat study 2, μFePP and high ratio CA and TSC FePP groups significantly increased hepatic iron concentrations compared with the high ratio CA and TSC FePO_4_ and control groups. In the cell-culture study, among coextruded samples, the ferritin concentrations of high ratio samples were significantly higher than those of low and zero ratio samples. Among mixed samples, the high ratio FePO_4_ had significantly higher ferritin concentration than the zero ratio FePO_4_.

**Conclusions:**

The iron bioavailability of FePP and FePO_4_ was improved with CA and TSC at a high molar ratio. High ratio FePP and μFePP were superior to high ratio FePO_4_ in increasing hepatic iron concentrations.

## Introduction

The largest contributor to anemia worldwide is iron-deficiency anemia (IDA), which is caused by inadequate iron for hemoglobin (Hb) synthesis [[1], [2], [3]]. Iron fortification and supplementation are promising approaches to combat many cases of iron-deficiency diseases, specifically those in high-risk groups [1,2,4]. A recent systematic review and meta-analysis, including 37 intervention-control comparisons, reported that iron-fortified salt had positive effects on Hb concentrations [5].

Rice is a staple food for half of the world’s population [6]. Rice can be fortified with iron to combat IDA, but it is challenging to achieve high iron bioavailability and good organoleptic properties. Iron can react with starch molecules to form a ferric starch complex through a coordinate bond C=O [7], and rice is sensitive to color changes when mixed with iron fortificants [8]. In general, common rice iron fortificants, ferric pyrophosphate (FePP) and ferric orthophosphate (FePO_4_), avoid discoloring white rice but have low bioavailability. Thus, iron absorption enhancers, food processing strategies, and micronizing iron fortificants are approaches used in an effort to improve the iron bioavailability [9].

Extrusion is a commercial food processing technology where food is cooked, squeezed, and gelatinized by the mechanical and thermal energy of the extruder, which can improve mineral bioavailability [10]. The viscosity, hardness, crystallinity, and other physicochemical properties of rice flour are changed during extrusion, including the disruption of the starch crystalline when it is degraded and gelatinized. Water absorption and extruded rice flour solubility are increased and inhibitors (e.g., phytic acid and phenolics) can be destroyed [11,12]. Thus, extruded rice flour, instead of raw rice flour or cooked rice flour, when mixed with iron and enhancers, improves iron digestibility [13]. In this study, the term “coextrusion” describes food processing that includes several micronutrients extruded concurrently [14]. However, it should be noted that some use “coextrusion” to refer to 1 staple food extruded with another staple food, fat-based (e.g., cream), or/and water based (e.g., jellies and jams) simultaneously [15].

Citric acid (CA) and trisodium citrate (TSC) dihydrate are readily absorbed in the gastrointestinal tract as an energy resource for the human body. CA and TSC can react with Fe^3+^ to generate an anticaking iron complex, ferric citrate (FeC_6_H_5_O_6_Na). They can prevent iron polymerization through their carboxylic bonds and hydroxyl bonds. Ferric citrate has good bioavailability and is generally recognized as safe for use in food aid. Thus, CA and TSC can work as enhancers of iron bioavailability by reacting with iron to generate ferric citrate complexes [[16], [17], [18], [19], [20], [21], [22]].

Chemically, FePP is composed of multiple orthophosphate groups with the P-O-P linkages (high-energy phosphate bond) and 3O^–^-Fe^3+^ coordination bonds. In a clinical trial, the iron absorption of ^57^FePP, CA, and TSC coextrusion was higher than that of extruded rice flour mixed with ^57^FePP, CA, and TSC [14]. Moreover, the iron bioavailability of rice flour fortified and extruded with ^57^FePP, CA, and TSC at a molar ratio of 1:0.1:2.1 was significantly higher than that of ^57^FePP rice extrudate without CA or TSC in iron-sufficient young females [9]. A higher molar ratio of enhancer (Fe:CA:TSC = 1:0.3:5.5) sharply increased the iron absorption of extruded rice fortified with ^57^FePP in iron-insufficient young females [13]. Extrusion with enhancers may transform the insoluble FePP into soluble ferric citrate complexes [13]. Theoretically, micronized iron fortificants have higher iron bioavailability compared with the regular size counterparts, because micronizing increases surface area so that they can be more easily solubilized in the gastrointestinal tract [[23], [24], [25]].

Isotopic iron studies, based on a single meal after an overnight fast, may overestimate iron bioavailability [26,27]. Compared with a daily diet fortified with iron, isotopically labeled iron may not determine the relative effectiveness of different iron fortificants as they are typically utilized. Therefore, we examined the iron bioavailability of regular FePP and FePO_4_ formulations using the rat prophylactic-preventative model and the in vitro INFOGEST digestion/Caco-2 cell model.

Four factors that may impact iron bioavailability were investigated. They were as follows: *1*) type of iron (FePP or FePO_4_), *2*) the particle size (regular or micronized), *3*) the molar ratio of enhancers (high or low ratio), and *4*) the food processing strategy (coextruded or mixed with enhancers) [9,13,14]. The objective is to determine the most effective fortification formulations to better inform iron rice fortification.

## Methods

### Materials

CA and TSC were purchased from Sigma-Aldrich. FePP, micronized FePP (μFePP), and FePO_4_ were kindly provided by Wright Enrichment Inc. Rice flour was produced by RIVLAND Partnership.

In 2 rat studies, animals were obtained from Charles River. Monoglyceride was produced by DuPont Danisco Food Ingredients, and soy protein isolate was supplied by Know-How Foods. American Institute of Nutrition (AIN)–93G rat diet, casein, cellulose, soybean oil, L-Cystine, mineral mix, and vitamin mix were purchased from Dyets, Inc.

In the cell-culture study, inserts were obtained from Fisher Scientific and silicone O-Rings were supplied by Uxcell. All other materials were procured in accordance with the INFOGEST protocol [28] and Dr. Raymond P. Glahn’s Caco-2 cell-culture method [29].

### Food processing strategies

Three strategies of food processing were performed to determine their impact on rice flour iron bioavailability.

#### Strategy 1 (coextruded, rat study 1)

Raw rice flour, vitamin-mineral mix, salt, monoglycerides, and iron were premixed (Table 1) and then extruded in a pilot-scale (large size) twin-screw extruder (Supplementary Table 1, Supplementary Figure 1). In the pilot-scale extruder, the raw rice flour was introduced to the gravimetric feed system, mixed by the high-intensity preconditioner, heated by recycled steam, and extruded by the mechanical energy of the screw rotating inside the barrel. The downspout from the preconditioner to the extruder helped reduce the fugitive dust into the environment. The knife hood could reduce clumps (Supplementary Figure 2). The final moist product exiting from the extruder die was pneumatically delivered to the dryer inlet. The extrudate was dried by a gas-fired dryer for 18 min with a 10-min cooling, and ground by a hammer mill with a mesh size of 0.3 mm. Because iron and rice flour were extruded concurrently by a pilot-scale extruder, this final product was called a coextruded (pilot-scale) rice flour sample (Supplementary Figure 1).

**TABLE 1:** Formulations of premixtures in the rat studies

Fe component	Fe compoundconcentration(mg/kg)	Enhancercompound	Enhancerconcentration(mg/kg)	Molar ratio(Fe:enhancer)	Molarratiolevel	Foodprocessingmethod	Other additives (mg/kg)		
							Vitamin-mineralmix	Monoglycerides	Salt
FePO_4_	130	None	None	None	Zero	Coextruded(strategy 1)	10	7.5	5
FePP	160	None	None	None	Zero	Coextruded(strategy 1)	10	7.5	5
μFePP	160	None	None	None	Zero	Coextruded(strategy 1)	10	7.5	5
FePP	140	CA	36	1:0.3	High	Coextruded(strategy 2)	None	None	None
		TSC	1014	1:5.5					
FePO_4_	121	CA	36	1:0.3	High	Coextruded(strategy 2)	None	None	None
		TSC	1014	1:5.5					
μFePP	140	None	None	None	Zero	Mixed (strategy 3)	None	None	None

No. 1–3 samples were designed for rat study 1. No. 4–6 samples were designed for rat study 2. Vitamin-mineral mix contained 0.11 IU vitamin A, 5.6 mg niacinamide, 3.5 mg zinc, 0.6 mg pyridoxine HCl, 0.47 mg thiamine mononitrate, 0.15 mg folic acid, and 1.1 mcg vitamin B12 per gram mix.

Abbreviations: CA, citric acid; Fe, iron; FePO_4_, ferric orthophosphate; FePP, ferric pyrophosphate; μFePP, micronized ferric pyrophosphate; TSC, trisodium citrate.

#### Strategy 2 (coextruded, rat study 2, and cell-culture study)

Raw rice flour, iron, CA, and TSC were premixed at a high molar ratio (1:0.3:5.5) or low molar ratio (1:0.1:2.1) (TABLE 1, TABLE 2) and extruded together by a lab-scale (small size) twin-screw extruder (Supplementary Table 1). In the laboratory-scale extruder, the raw rice flour was transferred into the feeder, which uniformly fed the flour into the extruder barrel that had several zones at different temperatures ranging from low to high, so the raw rice flour could be cooked and squeezed out by the twin-screw movement (Supplementary Figure 3). Extrudates were dried in the small-size oven at 70°C for 2 h, and manually cut to 1 cm. The extrudates were further ground by the coffee grinder, sifted to a final particle size below 1 mm, and frozen until use. Because rice flour, iron, CA, and TSC were extruded concurrently by a laboratory-scale extruder, this final product was called a coextruded (laboratory-scale) rice flour sample (Supplementary Figure 1).

**TABLE 2:** Sample design and iron fortification levels (700 mg/kg) for the cell-culture study

Fe component	Fe compoundconcentration(mg/kg)	AnalyticalFe elementconcentration(mg/kg)	Fe enhancercompound	Enhancerconcentration(mg/kg)	Molar ratio(Fe: enhancer)	Molarratiolevel	Foodprocessingmethod
FePP	2820	713	CA	720	1:0.3	High	Coextruded(strategy 2)
			TSC	20280	1:5.5		
FePP	2820	725	CA	240	1:0.1	Low	Coextruded(strategy 2)
			TSC	7740	1:2.1		
FePO_4_	2420	700	CA	720	1:0.3	High	Coextruded(strategy 2)
			TSC	20280	1:5.5		
FePO_4_	2420	688	CA	240	1:0.1	Low	Coextruded(strategy 2)
			TSC	7740	1:2.1		
FePP	2820	823	CA	720	1:0.3	High	Mixed(strategy 3)
			TSC	20280	1:5.5		
FePP	2820	767	CA	240	1:0.1	Low	Mixed(strategy 3)
			TSC	7740	1:2.1		
FePO_4_	2420	673	CA	720	1:0.3	High	Mixed(strategy 3)
			TSC	20280	1:5.5		
FePO_4_	2420	625	CA	240	1:0.1	Low	Mixed(strategy 3)
			TSC	7740	1:2.1		
μFePP	2820	875	CA	720	1:0.3	High	Mixed(strategy 3)
			TSC	20280	1:5.5		
μFePP	2820	910	CA	240	1:0.1	Low	Mixed(strategy 3)
			TSC	7740	1:2.1		
FePP	2820	683	None	None	None	Zero	Mixed (strategy 3)
FePO_4_	2420	616	None	None	None	Zero	Mixed (strategy 3)
μFePP	2820	927	None	None	None	Zero	Mixed (strategy 3)

The iron concentrations were assessed by the Soil Testing Lab, Kansas State University, Manhattan, KS, United States.

Abbreviations: CA, citric acid; Fe, iron; FePO_4_, ferric orthophosphate; FePP, ferric pyrophosphate; μFePP, micronized ferric pyrophosphate; TSC, trisodium citrate.

#### Strategy 3 (mixed, rat study 2, and cell-culture study)

Raw rice flour was extruded by a pilot-scale twin-screw extruder (Supplementary Table 1), dried by a gas-fired dryer at 115°C for 18 min with a 7-min cooling, and ground by a hammer mill with a mesh size of 0.3 mm. Subsequently, iron, CA, and TSC were mixed with extruded rice flour at a high/low/zero molar ratio (TABLE 1, TABLE 2) and frozen until use. Because iron, CA, and TSC were only mixed with the extruded rice flour, this final product was called a mixed extruded rice flour sample (Supplementary Figure 1).

### Food preparation

In rat study 1, the coextruded (pilot-scale, TX-52, Wenger Manufacturing) premixed rice flour was included in the final diet at a 1% weight ratio, with 84.5% natural white rice, 10.5% soy protein isolate, and 4% soybean oil to meet the protein and lipid requirements of growing rats [30]. The rice diets were cooked in an electric rice cooker with an equal part of water to achieve an added moisture content of ∼40%, which was further reduced during cooling. A goal of 35% added moisture content was determined based on studies evaluating water addition levels on protein efficiency in rats [31], and the maximum observed water addition before intake was decreased in female weanling rats [30]. The target level of iron fortification was 45 mg/kg rice flour. Diets were prepared twice weekly and stored in a 4°C refrigerator until use for the rat diet. The analytical data indicated that rice diets contained 15.2 g protein, 4 g fat, 11 g moisture, 0.6 g ash, and 1.41 mg iron per 100 g rice flour (Supplementary Table 2). Moisture content was calculated from the initial moisture of dry ingredients, the moisture of freshly prepared cooked diets, and moisture loss after 24 h at ambient conditions. The mean calculated total moisture of the cooked diets was 42%, which was 7% higher than the target of 35%.

In rat study 2, high ratio FePP/FePO_4_ coextruded (laboratory-scale, Micro-18, American Leistritz Extruder Corp.) and zero ratio FePP/μFePP mixed rice flour products (5% premixture) were blended with 20% casein, 5% cellulose, 7% soybean oil with tertiary-butylhydroquinone, 3.5% mineral mix, 1% vitamin mix, 0.3% L-Cystine, and 58.2% extruded rice flour that was produced using strategy 2 in line with the AIN-93G composition. The iron concentration of AIN-93G was 45 mg/kg food. All major mixing products (final rat diets) were prepared and stored at –80°C 1 wk before the beginning of the rat study. They were gradually transferred to a 4°C refrigerator for use. The analytical data indicated that the final rat diets contained 21.7 to 22.8 g protein, 6.17 to 6.28 g fat, 7.27 to 7.37 g moisture, 0.95 to 1.39 g fiber, 3.39 to 3.49 g ash, and 9.26 to 11.8 mg iron per 100 g rice flour (Supplementary Table 2).

In the cell-culture study, 13 rice flour samples were designed. They were high/low ratio coextruded FePP/FePO_4_ samples and high/low/zero ratio mixed FePP/μFePP/FePO_4_ samples. The level of iron fortification in premixtures was 700 mg/kg rice flour (Table 2). Because the final target level of iron fortification recommended by USDA was 40 mg/kg rice flour, the coextruded rice flour samples and mixed extruded rice flour samples were diluted with extruded rice flour that was produced using strategy 2 (Supplementary Table 3). This study was split into 2 experiments. In experiment 1, the iron solubility of premixtures was measured. The objective of experiment 2 was to assess the iron solubility and bioavailability of formulations. Samples were stored at –80°C until use.

### Animal experimental protocol

The animal experimental protocols were approved by the Institutional Animal Care and Use Committee at Kansas State University. The intended study duration (28 d) and size were based on the preventative prophylactic [32] and protein efficiency ratio methods [33] and our previous work using these models [32,33]. Male weanling Sprague-Dawley rats were randomly assigned into 10 animals per diet group and were housed individually in wire-bottom cages, provided with a resting board, tongue depressors as enrichment, and ad libitum access to the food and water. The control group was fed an AIN-93G diet [34]. Food intake of each rat was calculated from the food remnants and the fresh diet provided afterward. The weight of the rats was collected at baseline upon arrival, and subsequently measured every other day and weekly. After killing with carbon dioxide (CO_2_) inhalation along with cardiac puncture for blood collection, liver tissues were harvested, weighed, and flash-frozen in liquid nitrogen and stored at –80°C. Samples were wet ashed as previously described [35] and analyzed by inductively coupled plasma–optical emission spectrometry (ICP-OES; Varian 720-ES, Agilent Technologies) at the Kansas State University Soil Testing Lab. Rat body scans were performed on the rats’ carcasses using the dual-energy x-ray absorptiometry PIXImus densitometer (GE Lunar Corporation) for body composition and bone mineral density detection.

In rat study 1, the rats were killed on day 21. Hb concentration was quantified with QuantiChrom Whole Blood Hb Kit (DWHB-250, BioAssay Systems Hayward). Moisture adjustments were calculated for food intake, energy efficiency, and protein efficiency outcomes based on a 6.6% moisture basis, the moisture content of AIN-93G [34].

In rat study 2, the rats were killed on day 28; Hb concentration, hematocrit, and red blood cell indices were measured in the fresh whole blood added into tripotassium EDTA tubes (Fisher Scientific) for flow cytometry.

### INFOGEST procedure

We recently investigated using this method to determine the digestion of corn samples [36]. More detailed methods are found in it, but initially, electrolyte solutions, simulated salivary fluid, simulated gastric fluid, and simulated intestinal fluid were prepared and used for all digestion phases [28]. Manufacturers recommend reassessing enzyme activities when the products are stored for >3 y. As all reagents used in this study were newly purchased and stored for <1 y, the enzyme activity values provided by the manufacturer (Sigma-Aldrich) were used directly. The iron content of dry samples and supernatant was analyzed with the ICP-OES method by the Soil Testing laboratory at Kansas State University. Solubility (%) = 100 × soluble iron content/total iron content [28,[37], [38], [39]].

### Caco-2 cell-culture procedure

We recently investigated using this method to determine the iron bioavailability of corn samples [36]. More detailed methods are found in it, but Caco-2 cells were purchased from American Type Culture Collection at passage 18. The Caco-2 cells were cultured in T25, T75, and T225 flasks with Dulbecco's modified Eagle's medium supplemented with 10% fetal bovine serum, 1% Zellshield, and 25 mM HEPES, and incubated at 37°C and in 5% CO_2_ air [29].

On day 12, media were changed before day 13. Two mL of minimum essential medium (MEM) was replaced by 1 mL of MEM in the lower chamber, and 1.5 mL of iron digesta in the upper chamber. Iron treatments were randomly assigned to the Caco-2 cells by a table generated with Microsoft Excel. The 6-well plates were placed on a rocking shaker (6 oscillations/min) for 2 h. The insert frame and dialysis membrane were removed, and 1 mL of MEM was added. The Caco-2 cells were returned to the incubator for another 22 h. A control (only media) was used as a reference.

**FIGURE 1:**
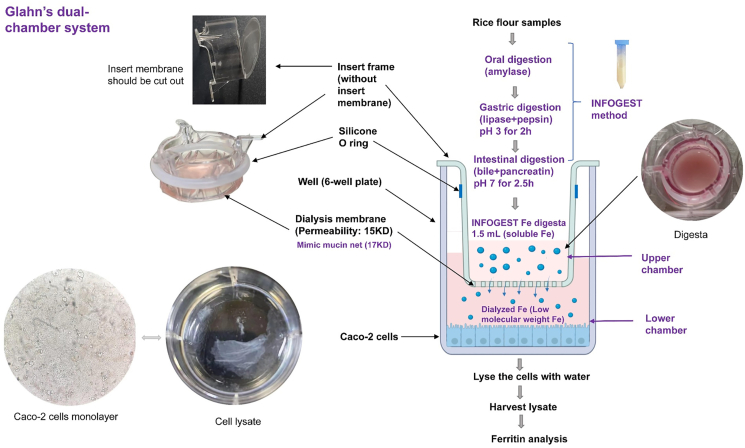
Glahn’s dual-chamber system.

On the 14th d, MEM was removed, and autoclaved deionized water was added to osmotically lyse the Caco-2 cells [29]. Then the 6-well plates were placed on the tube rack in a sonicator for 30 s to further lyse the cells [40]. Finally, cell lysate was collected for ferritin and protein analyses according to the instructions of the ferritin and protein kits (Figure 1) [29]. Ferritin concentrations of premixtures were not assessed with the Caco-2 cell model due to a potential risk of iron overload.

### Statistical analysis

Data were analyzed in SAS Online Studio (https://welcome.oda.sas.com/) with significance at *P* value of <0.05. The Shapiro–Wilk test was used for normality, and Levene’s test for homogeneity. Data were analyzed by 1-way analysis of variance with the least significant difference test. ELISA analysis was performed using the Arigo online calculator at https://www.arigobio.com/elisa-analysis.

## Results

### Rat study 1: amino acid levels

The concentrations of lysine, available lysine, and cysteine + methionine were 7.98, 7.76, and 5.15 mg/g rice flour, respectively.

### Rat studies: food intake and morphometric evaluation

In rat study 1, the adjusted food intakes, adjusted caloric efficiency, adjusted protein efficiency, weight gains, final weights, and bone mineral densities of all iron-fortification groups were significantly lower than those in the AIN-93G group (Supplementary Figure 4, Table 3). There were no significant differences in lean mass among all groups.

**TABLE 3:** Rat study 1: food intake, food efficiencies, and morphometric outcomes

	AIN-93G	FePO_4_	FePP	μFePP
Total food intake (g)	338.4 ± 30.5^a^	295.5 ± 30.1^ab^	307.9 ± 33.4^ab^	282.5 ± 30.8^b^
Adjusted total food intake (g)	338.4 ± 30.5^a^	191.1 ± 19.5^b^	199.1 ± 21.6^b^	182.7 ± 19.9^b^
Total weight gain (g)	171.3 ± 16.1^a^	23.2 ± 4.7^b^	25.7 ± 6.3^b^	21.2 ± 4.3^b^
Final body weight (g)	229.7 ± 23.0^a^	83.2 ± 9.7^b^	83.1 ± 9.8^b^	81.0 ± 7.1^b^
Caloric efficiency (g/100 kcal)	127.8 ± 3.3^a^	20.9 ± 3.3^b^	22.3 ± 4.5^b^	19.9 ± 3.1^b^
Adjusted caloric efficiency (g/100 kcal)	127.8 ± 3.3^a^	32.3 ± 5.1^b^	34.4 ± 6.9^b^	30.8 ± 4.8^b^
Protein efficiency (g/10 g)	25.3 ± 0.6^a^	5.1 ± 0.8^b^	5.5 ± 1.1^b^	4.9 ± 0.8^b^
Adjusted protein efficiency (g/10 g)	25.3 ± 0.6^a^	7.9 ± 1.3^b^	8.5 ± 1.7^b^	7.6 ± 1.2^b^
Lean mass (%)	89.2 ± 1.1	90.1 ± 1.0	90.5 ± 0.9	89.9 ± 0.8
Bone mineral density (g/cm^2^) × 1000	84.3 ± 6.5^a^	44.3 ± 1.8^b^	44.3 ± 3.6^b^	44.2 ± 4.1^b^

Data were represented as mean ± SD, *n* = 10; values in the same row with different superscripts were significantly different at *P* < 0.05.

Abbreviations: AIN, American Institute of Nutrition; FePO_4_, ferric orthophosphate; FePP, ferric pyrophosphate; μFePP, micronized ferric pyrophosphate.

In rat study 2, there were no significant differences in the weekly food intake, total food intake, weight gain, mean weekly weights, final body weights, lean mass, and bone mineral density among all groups (Supplementary Figures 5, Table 4).

**TABLE 4:** Rat study 2: food intake and morphometric outcomes

	AIN-93G	μFePP	High FePO_4_	High FePP
Total food intake (g)	559.64 ± 9.51	551.53 ± 13.14	552.55 ± 13.45	515.14 ± 21.22
Total weight gain (g)	251.68 ± 5.55	264.04 ± 6.09	263.86 ± 6.26	239.65 ± 9.03
Final body weight (g)	310.63 ± 7.87	324.32 ± 8.35	322.51 ± 6.65	298.63 ± 11.00
Lean mass (%)	89.65 ± 0.55	90.96 ± 0.33	90.54 ± 0.42	89.95 ± 0.43
Bone mineral density (g/cm^2^) ×1000	113.82 ± 2.40	112.13 ± 3.26	112.91 ± 2.16	108.09 ± 2.91

Data were represented as mean ± SEM, *n* = 10.

Percentage lean mass: Total weight minus fat mass divided by total weight × 100.

Food intake was measured every other day by subtracting food remnants from the previous food given.

Abbreviations: AIN, American Institute of Nutrition; FePO_4_, ferric orthophosphate; FePP, ferric pyrophosphate; μFePP, micronized ferric pyrophosphate.

### Rat studies: hematological and hepatic iron outcomes

In rat study 1, Hb and hepatic iron levels were significantly higher in the FePP and μFePP groups compared with the FePO_4_ and control groups (Table 5).

**TABLE 5:** Rat study 1: hematological and hepatic iron outcomes

	AIN-93G	FePO_4_	FePP	μFePP
Hemoglobin (g/dL)	12.5 ±1.0^c^	13.3 ± 2.3^bc^	14.9 ± 0.8^a^	15.0 ± 1.0^a^
Hepatic Iron (μg/g)	8.7 ± 2.0^b^	10.0 ± 1.5^b^	16.0 ± 4.8^a^	14.6 ± 4.0^a^

Data were represented as mean ± SD, *n* = 10; values in the same row with different superscripts were significantly different at *P* < 0.05.

Abbreviations: AIN, American Institute of Nutrition; FePO_4_, ferric orthophosphate; FePP, ferric pyrophosphate; μFePP, micronized ferric pyrophosphate.

In rat study 2, there were no significant differences in the Hb concentration, hematocrit, red blood cell count, mean cell Hb, mean cell Hb concentration, mean cell volume, and total white blood cell counts between groups (Table 6). Hepatic iron concentrations of μFePP and High FePP groups were significantly increased compared with High FePO_4_ and control groups.

**TABLE 6:** Rat study 2: hematological and hepatic iron outcomes

	AIN-93G	μFePP	High FePO_4_	High FePP
Hematocrit (%)	49.1 ± 0.7	50.1 ± 0.9	49.8 ± 0.7	50.5 ± 0.6
Hemoglobin concentration (g/dL)	13.8 ± 0.2	14.3 ± 0.3	14.0 ± 0.2	14.4 ± 0.2
Red blood cell counts (M/μL)	6.9 ± 0.1	6.9 ± 0.1	7.1 ± 0.1	7.0 ± 0.1
Mean cell volume (fl)	71.0 ± 1.1	73.1 ± 0.7	69.9 ± 1.7	72.4 ± 0.9
Mean cell hemoglobin (pg)	20.0 ± 0.3	20.8 ± 0.2	19.6 ± 0.5	20.6 ± 0.3
Mean cell hemoglobin concentration (g/dL)	28.18 ± 0.10	28.52 ± 0.20	28.06 ± 0.20	28.50 ± 0.25
Total white blood cell count (K/μL)	12.74 ± 0.80	12.60 ± 0.60	11.81 ± 0.90	13.84 ± 1.10
Hepatic iron (μg/g)	7.1 ± 0.5^a^	11.4 ± 1.3^b^	8.1a ± 0.4^a^	11.5b ± 1.2^b^

Data were represented as mean ± SEM, *n* = 10; values in the same row with different superscripts were significantly different at *P* < 0.05.

Abbreviations: AIN, American Institute of Nutrition; FePO_4_, ferric orthophosphate; FePP, ferric pyrophosphate; μFePP, micronized ferric pyrophosphate.

### Cell-culture study: supernatant iron solubility

**FIGURE 2:**
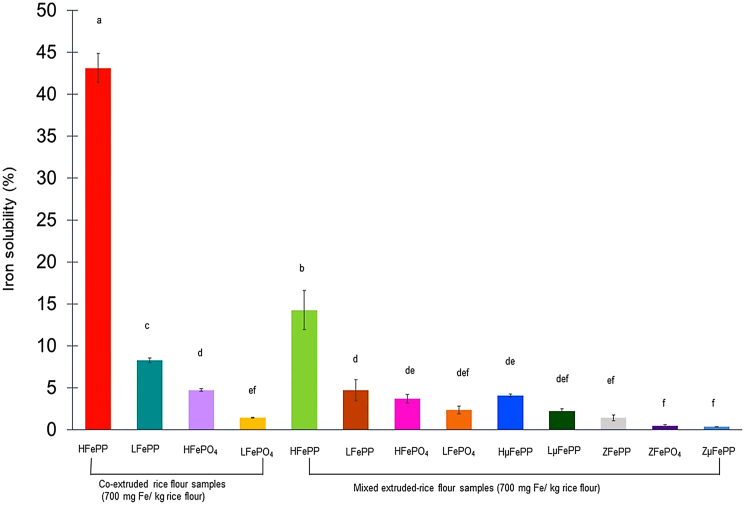
Iron solubility of Fe-fortified samples at 700 mg/kg level. Data were mean ± SEM, *n* = 3; values with different letters were significantly different at *P* < 0.05. FePO_4_, ferric orthophosphate; FePP, ferric pyrophosphate; μFePP, micronized ferric pyrophosphate.

In experiment 1, iron solubilities of coextruded high and low FePP samples were significantly higher than mixed high and low FePP samples (Table 7, Figure 2). Among coextruded samples (Table 7, Figure 2), the iron solubilities of high and low FePP samples were significantly higher than those of high and low FePO_4_ samples. Among coextruded samples (Table 7, Figure 2), high FePP and high FePO_4_ samples had iron solubilities that were significantly higher than low FePP and low FePO_4_ samples. Among mixed samples, the high FePP sample had higher iron solubility than low FePP, high FePO_4_, high μFePP, and zero FePP samples. Among mixed samples, high and low FePO_4_/μFePP samples were significantly higher than zero FePO_4_/μFePP samples, respectively.

**TABLE 7:** Iron solubility (experiment 1700mg Fe/kg level)

Fe component	Molar ratio level	Food processing method (premixture)	Iron solubility (%)
FePP	High	Coextruded	43.1 ± 1.72^a^
FePP	Low	Coextruded	8.3 ± 0.27^c^
FePO_4_	High	Coextruded	4.7 ± 0.16^d^
FePO_4_	Low	Coextruded	1.4 ± 0.04^ef^
FePP	High	Mixed	14.3 ± 2.34^b^
FePP	Low	Mixed	4.7 ± 1.25^d^
FePO_4_	High	Mixed	3.7 ± 0.50^de^
FePO_4_	Low	Mixed	2.4 ± 0.46^def^
μFePP	High	Mixed	4.1 ± 0.18^de^
μFePP	Low	Mixed	2.2 ± 0.29^def^
FePP	Zero	Mixed	1.4 ± 0.34^ef^
FePO_4_	Zero	Mixed	0.5 ± 0.15^f^
μFePP	Zero	Mixed	0.4 ± 0.01^f^
Control	None	None	None

Data were represented as mean ± SEM, *n* = 3; values in the same row with different superscripts were significantly different at *P* < 0.05.

Abbreviations: FePO_4_, ferric orthophosphate; FePP, ferric pyrophosphate; μFePP, micronized ferric pyrophosphate.

**FIGURE 3:**
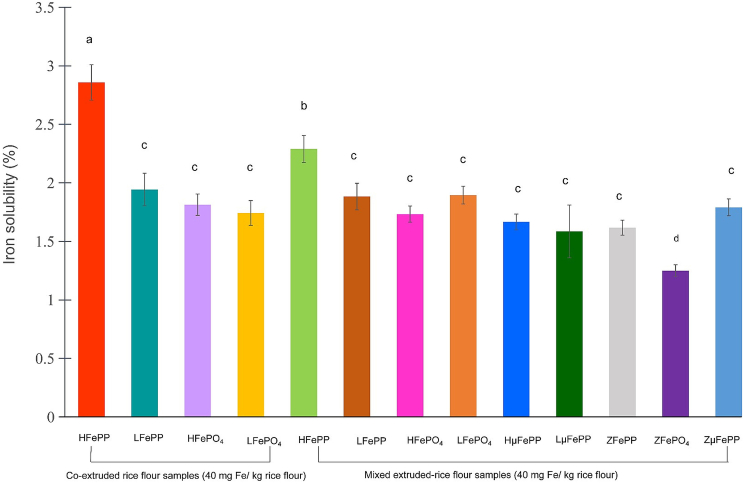
Iron solubility of Fe-fortified samples at 40 mg/kg level. Data were mean ± SEM, *n* = 3; values with different letters were significantly different at *P* < 0.05. HFePP, High citric acid/trisodium citrate concentration ferric pyrophosphate; LFePP, Low citric acid/trisodium citrate concentration ferric pyrophosphate; HFePO__4__, High citric acid/trisodium citrate concentration ferric orthophosphate; LFePO__4__, Low citric acid/trisodium citrate concentration ferric orthophosphate; HμFePP, High citric acid/trisodium citrate concentration micronized ferric pyrophosphate; LμFePP, Low citric acid/trisodium citrate concentration micronized ferric pyrophosphate; ZFePP, Zero citric acid/trisodium citrate ferric pyrophosphate; ZHFePO__4__, Zero citric acid/trisodium citrate ferric orthophosphate; ZμFePP, Zero citric acid/trisodium citrate micronized ferric pyrophosphate.

In experiment 2, the iron solubility of the coextruded high FePP sample was significantly higher than that of the mixed high FePP sample (Table 8, Figure 3). Among coextruded samples (Table 8, Figure 3), the high FePP sample had higher iron solubility than the low FePP and high FePO_4_ samples. Among mixed samples (Table 8, Figure 3), the high FePP sample had significantly higher iron solubility than the low FePP, high FePO_4_, high μFePP, and zero FePP samples, respectively. High and low FePO_4_ samples had higher iron solubility than the zero FePO_4_ sample.

**TABLE 8:** Iron solubility and bioavailability (experiment 2, 40 mg Fe/kg level)

Fe component	Molar ratio level	Food processing method (premixture)	Iron solubility (%)	Ferritin concentration (ng/mg protein)
FePP	High	Coextruded	2.9 ± 0.15^a^	25.82 ± 3.82^a^
FePP	Low	Coextruded	1.9 ± 0.14^c^	12.44 ± 0.60^cd^
FePO_4_	High	Coextruded	1.8 ± 0.09^c^	25.46 ± 4.69^ab^
FePO_4_	Low	Coextruded	1.7 ± 0.01^c^	7.14 ± 0.99^de^
FePP	High	Mixed	2.3 ± 0.01^b^	18.87 ± 3.49^bc^
FePP	Low	Mixed	1.9 ± 0.01^c^	8.34 ± 0.90^de^
FePO_4_	High	Mixed	1.7 ± 0.07^c^	22.64 ± 3.96^ab^
FePO_4_	Low	Mixed	1.9 ± 0.08^c^	11.25 ± 0.96^cd^
μFePP	High	Mixed	1.7 ± 0.07^c^	12.90 ± 0.99^cd^
μFePP	Low	Mixed	1.6 ± 0.02^c^	4.63 ± 0.29^e^
FePP	Zero	Mixed	1.6 ± 0.06^c^	6.56 ± 1.59^de^
FePO_4_	Zero	Mixed	1.3 ± 0.05^d^	3.05 ± 0.54^e^
μFePP	Zero	Mixed	1.8 ± 0.07^c^	4.47 ± 1.08^e^
Control	None	None	None	3.92 ± 0.67^e^

Data were represented as mean ± SEM, *n* = 3; values in the same row with different superscripts were significantly different at *P* < 0.05.

Abbreviations: FePO_4_, ferric orthophosphate; FePP, ferric pyrophosphate; μFePP, micronized ferric pyrophosphate.

### Cell-culture study of iron bioavailability (ferritin formation)

**FIGURE 4:**
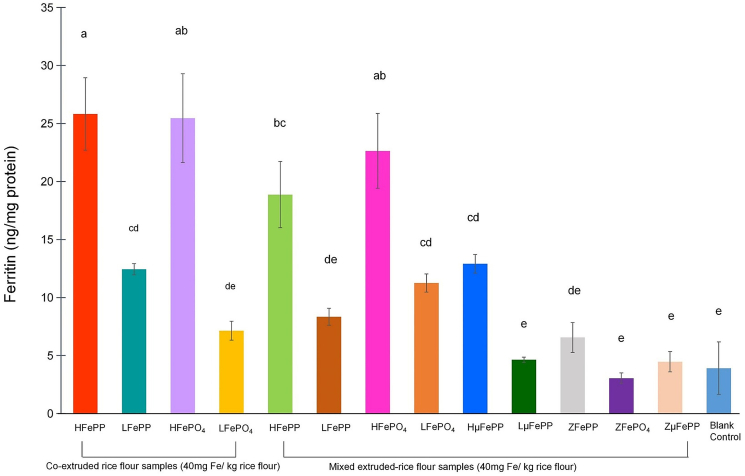
Iron bioavailability of Fe-fortified samples at 40 mg/kg level. Data were mean ± SEM, *n* = 3; values with different letters were significantly different at *P*< 0.05. HFePP, High citric acid/trisodium citrate concentration ferric pyrophosphate; LFePP, Low citric acid/trisodium citrate concentration ferric pyrophosphate; HFePO__4__, High citric acid/trisodium citrate concentration ferric orthophosphate; LFePO__4__, Low citric acid/trisodium citrate concentration ferric orthophosphate; HμFePP, High citric acid/trisodium citrate concentration micronized ferric pyrophosphate; LμFePP, Low citric acid/trisodium citrate concentration micronized ferric pyrophosphate; ZFePP, Zero citric acid/trisodium citrate ferric pyrophosphate; ZHFePO__4__, Zero citric acid/trisodium citrate ferric orthophosphate; ZμFePP, Zero citric acid/trisodium citrate micronized ferric pyrophosphate.

In experiment 2, among coextruded samples (Table 8, Figure 4), high FePP, high FePO_4_ ferritin concentrations were significantly higher than low FePP, low FePO_4,_ and the control. Among mixed samples (Table 8, Figure 4), high FePP, high FePO_4_, and high μFePP ferritin concentrations were significantly higher than low/zero FePP, low/zero FePO_4,_ low/zero μFePP, and the control. Coextruded low FePP and mixed low FePO_4_ ferritin concentrations were significantly higher than the control.

## Discussion

In rat study 1, the notable limitation was that low food intake led to poor growth. Thus, the study duration was shortened to 3 wk from the intended 4 wk. Multiple possibilities might be useful in explaining this limitation. First, vitamin and mineral mixes should account for 1% of the final products rather than the premixtures. Moreover, the quantity and quality of protein might be low. The biological utilization of soybean protein is inferior to that of casein [41]. Third, the fluid in the diets designed to mimic human cooking and feeding practices may not be feasible for rats to meet caloric needs.

Compared with the cooked natural rice in rat study 1, extruded rice flour in rat study 2 was well-received by the rats as an appropriate staple food for delivering micronutrients.

In rat study 2, the iron treatment groups were fortified at higher levels than the AIN group, explaining the higher iron outcomes. However, compared with high FePO_4,_ high FePP, and zero μFePP, improvements had statistical and biological significance, as they had similar iron fortification levels. Extruded rice cofortified with FePP, CA, and TSC has been demonstrated to enhance iron absorption in iron-replete young females [9,13]. Likewise, consumption of μFePP-fortified extruded rice (18 mg Fe/d) for 8 mo has been reported to improve iron stores and reduce the prevalence of IDA among Indian school children aged 5 to 11 y [42].

However, μFePP (2.4 μm) was not superior to FePP (rat study 1) or high FePP (rat study 2) in Hb concentrations or hepatic iron concentrations, respectively. It might be because μFePP was not coextruded with enhancers and rice flour. In line with our results, there was no significant difference in the relative bioavailability value between μFePP (2.5 μm) and regular FePP (21 μm) in a rat study [43]. In an African clinical trial, the Hb concentrations of iron-deficient children aged 6 to 15 y were not improved by a 6-mo μFePP (2.5 μm, 10 mg Fe/d) treatment [44]. Another possible explanation was that the particle size of μFePP was still too large to improve bioavailability. It has been found that large μFePP (5.2 μm) had an inferior Hb gain to ferrous sulfate (FeSO_4_) in rats, whereas small μFePP (0.3 μm) was as effective as FeSO_4_ in Hb gain [45]. In a clinical trial, isotopic μFePP (0.3 μm) also had the equivalent iron absorption to FeSO_4_ in infant cereal and yogurt [25]. Thus, the relatively large particle size of μFePP (2.4 μm) may have contributed to its lack of significant bioavailability in our study.

In the cell-culture study, FePP and enhancers coextruded with rice flour at a high molar ratio had the highest iron solubility, but they did not improve the ferritin concentrations compared with FePO_4_. The reason might be that the Fe concentrations of coextrudated FePP, CA, and TSC (40 mg Fe/kg rice) were too low. In another study using coextruded FePP, CA, and TSC with an iron concentration of 80 mg Fe/kg rice, an increasing trend in iron solubility was observed with higher molar ratios of CA and TSC, and iron absorption from the coextruded meal with a molar ratio of 1:0.1:2.1 was equivalent to that from the FeSO_4_ reference meal [9]. The molar ratio of the enhancers is the meaningful contributor to iron bioavailability. In other Caco-2 cell studies, it was also found that CA could significantly increase the Fe^3+^ uptake [46,47]. Similarly, a Caco-2 cell experiment showed that CA could improve iron absorption in milk at a 1:1 molar ratio [48].

In addition, there was no significant difference between regular FePP, μFePP, and FePO_4_ in ferritin response. One possibility is that the amount of soluble iron in FePP digesta, μFePP digesta, and FePO_4_ digesta was equivalent. Although iron solubility from the supernatant served as a useful reference in the current study, the predictive ability of iron solubility for iron bioavailability is limited [49,50]. It may be more accurate to measure solubility from the digesta/dialysate, as the iron digesta/dialysate was applied to Caco-2 cells to assess ferritin responses. A positive correlation between dialyzed iron and iron absorption was observed in an in vitro digestion study [51].

Because the iron solubility of FePP samples at 700 mg Fe/kg level was much higher than that of FePP samples at 40 mg Fe/kg, another possibility was that the FePP iron bioavailability is improved at higher fortification levels. In 1 Caco-2 cell experiment using cereal fortified with 300 mg Fe/kg, sodium iron EDTA (NaFeEDTA) and ferrous fumarate showed significantly higher ferritin responses compared with FeSO_4_ [52]. In another Caco-2 cell experiment using bread fortified with 37 mg Fe/kg, the ferritin concentration from the NaFeEDTA sample was significantly lower than that from the FeSO_4_ sample, and the ferritin concentrations of ferrous fumarate samples were equivalent to that of the FeSO_4_ sample [53]. However, this perspective remains controversial. For example, the Brazilian food study reported no significant association between the iron concentration of by-products and predicted bioavailability, despite all food samples having high iron levels [54]. Another factor may be the sensitivity and accuracy of ICP-OES, because the minimum detectable concentration of iron is around 0.2 mg/L, whereas the iron concentrations of the samples’ supernatant ranged from 0.13 to 0.19 mg/L.

The present study has several design limitations. This study did not provide mechanistic explanations for the observed differences in iron bioavailability. The primary objective of the current study was to compare outcomes across formulations and processing conditions. FePP and FePO_4_ may differ in crystal structure, particle surface, solubility, food matrix interaction, extrusion effect, and CA/TSC effect, which can lead to different iron bioavailability. Mechanistic studies at the molecular level would strengthen the scientific depth of this work.

In conclusion, the iron bioavailability of FePP and FePO_4_ was improved with a high molar ratio (1:0.3:5.5) of CA and TSC. μFePP and high FePP were superior to high FePO_4_ in the hepatic iron bioavailability of rats. Therefore, formulations of μFePP/FePP with a high molar ratio of CA and TSC are promising for rice fortification. More research is needed to refine the iron formulations and food processing strategies.

## Author contributions

The authors’ responsibilities were as follows – BLL, SA: designed the research; SA, TG, MJ, MI: produced the extrudates, produced the viscosity data, and contributed food processing knowledge; JZ: conducted the cell-culture study and wrote the article; EJW, HARS: conducted rat study 1; DBA, JZ: conducted rat study 2; JZ, EJW, DBA, MJ, SA, BLL: revised the article; BLL: took primary responsibility for the final content; and all authors: read and approved the final manuscript.

## Data availability

The authors confirm that all data supporting the findings of this study are included within the article. SAS code book and analytic code are freely and publicly accessible without restriction at https://support.sas.com/documentation/onlinedoc/stat/131/anova.pdf and https://video.sas.com/detail/video/5537529513001/the-one-way-anova-task-in-sas-studio.

## Declaration of Generative AI and AI-assisted technologies in the writing process

The authors declare that no generative AI or AI-assisted technologies were used in the writing of this manuscript.

## Funding

USDA Micronutrient Fortified Food Aid Products Pilot Program, USDA Multistate 4002, and Kansas State Agriculture Experiment Station.

## Conflict of interest

The authors report no conflicts of interest.
