# Supplementary material for: Bioavailability of Ferric Pyrophosphate and Ferric Orthophosphate with/without Extrusion and/or Citric Acid and/or Trisodium Citrate in Rats and In Vitro INFOGEST Digestion and Caco-2 Cell Model
Source: Curr Dev Nutr. 2026 May 18;10(6):107718. doi: 10.1016/j.cdnut.2026.107718 (PMC13263688; doi:10.1016/j.cdnut.2026.107718)
Supplement: Multimedia component 1 [file mmc1.docx]

**Supplementary Table 1. Extruder Parameters**

| Parameter | Pilot-scale  (Strategy 1) | |  | Lab-scale  (Strategy 2) | Pilot-scale  (Strategy 3) |  |
| --- | --- | --- | --- | --- | --- | --- |
| Volumetric capacity (m^3^) | 0.056 |  | | Unknown | 0.056 |  |
| Preconditioner shaft speed (rpm) | 400 |  | | Not applicable | 379 |  |
| Average residence time (minute) | Unknown |  | | Unknown | 2.8 |  |
| Barrel temperature (°C) | 44-70 |  | | 40-90 | 25-90 |  |
| Screw diameter (mm) | 52 |  | | 18 | 52 |  |
| L/D ratio | 16 |  | | 29 | 16 |  |
| Feed speed (kg/h) | 60 |  | | 2.76 | 80 |  |
| Screw speed fixed (rpm) | 300 |  | | 350 | 300 |  |
| Circular die | 3.7 |  | | 3.1 | 3.7 |  |
| Knife blades rotating speed (rpm) | 2750 |  | | Cut by hand | 530 |  |

**Supplementary Table 2. Nutrient concentrations of final products and AIN-93G in the rat studies**

| **Components** | **AIN-93G** | **Nature rice diets in Rat Study 1** | **Final rat diets in Rat Study 2** | | |  |
| --- | --- | --- | --- | --- | --- | --- |
|  |  |  | µFePP | High FePO_4_ | High FePP | |
| Protein (g/100g) | 20.0 | 15.2 | 22.81 | 22.21 | 21.73 | |
| Fat (g/100g) | 7.00 | 4 | 6.19 | 6.28 | 6.17 | |
| Fiber (g/100g) | 5.00^*^ | 0 | 1.32 | 1.39 | 0.95 | |
| Moisture (g/100g) | 6.60 | 11 | 7.27 | 7.30 | 7.37 | |
| Ash (g/100g) | 4.17^*^ | 0.6 | 3.39 | 3.49 | 3.46 | |
| Iron (mg/100g) | 6.6^#^ | 1.41 | 11.80 | 9.26 | 10.80 | |

Note: The *values were collected from the product label of AIN-93G. The ^#^iron concentration was assessed by AIB International, Manhattan, KS. Others were measured by University of Missouri–Columbia Agricultural Experiment Station Chemical Laboratories (Columbia, MO).

**Supplementary Table 3. Sample design and iron fortification levels (40 mg/kg) for the cell-culture study**

| **Fe component** | **Food processing method (premixture)** | **Premixture concentration**  **(mg/100g)** | **Additional extruded rice flour (mg/100g)** | **Fe element concentration (mg/kg) (final product)** | **Enhancer molar ratio level** |  |
| --- | --- | --- | --- | --- | --- | --- |
|  |  |  |  |  |  |  |
| FePP | Co-extruded | 4.92 | 95.08 | 56.9 | High |  |
|  |  |  |  |  |  |  |
| FePP | Co-extruded | 5.06 | 94.94 | 48.7 | Low |  |
|  |  |  |  |  |  |  |
| FePO_4_ | Co-extruded | 4.26 | 95.74 | 54.2 | High |  |
|  |  |  |  |  |  |  |
| FePO_4_ | Co-extruded | 5.3 | 94.7 | 51.1 | Low |  |
|  |  |  |  |  |  |  |
| FePP | Mixed | 4.86 | 95.14 | 41.9 | High |  |
|  |  |  |  |  |  |  |
| FePP | Mixed | 5.22 | 94.78 | 47.3 | Low |  |
|  |  |  |  |  |  |  |
| FePO_4_ | Mixed | 5.94 | 94.06 | 50.2 | High |  |
|  |  |  |  |  |  |  |
| FePO_4_ | Mixed | 6.4 | 93.6 | 45.9 | Low |  |
|  |  |  |  |  |  |  |
| μFePP | Mixed | 4.58 | 95.42 | 52.2 | High |  |
|  |  |  |  |  |  |  |
| μFePP | Mixed | 4.4 | 95.6 | 56.9 | Low |  |
|  |  |  |  |  |  |  |
| FePP | Mixed | 5.86 | 94.14 | 50.2 | None |  |
| FePO_4_ | Mixed | 6.5 | 93.5 | 60.3 | None |  |
| μFePP | Mixed | 4.32 | 95.68 | 42.1 | None |  |

Note: The iron concentrations were assessed by Soil Testing Lab, Kansas State University, Manhattan, KS.

**
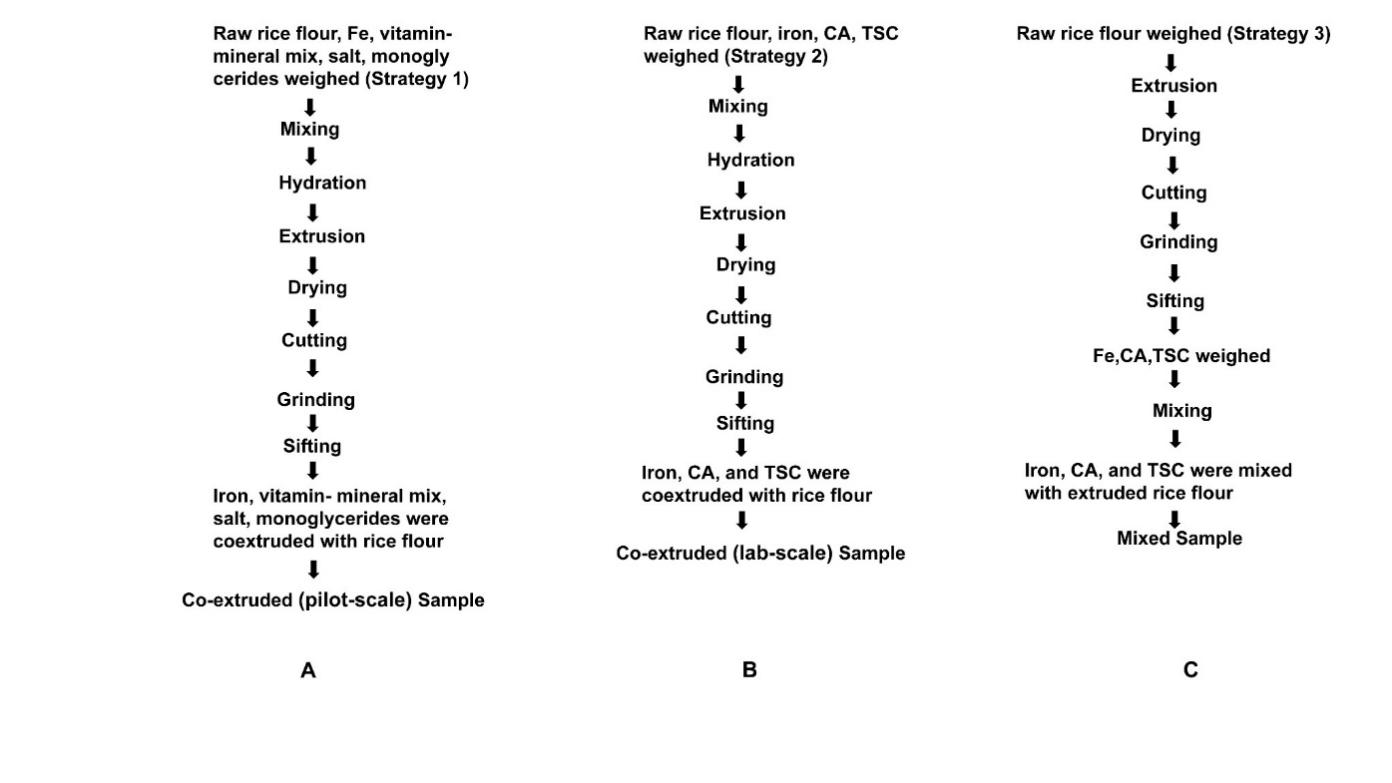
**

**Supplementary Figure 1: Flow chart of food processing strategies**

A: Food processing strategy 1. Iron and rice flour was extruded concurrently by pilot-scale extruder. This final product was called co-extruded (pilot-scale) rice flour sample. B: Food processing strategy 2. Rice flour, iron, CA and TSC were extruded concurrently by lab-scale extruder. This final product was called co-extruded (lab-scale) rice flour sample. C: Food processing strategy 3. Iron, CA, and TSC were only mixed with the extruded rice flour. This final product was called mixed extruded-rice flour sample.

**
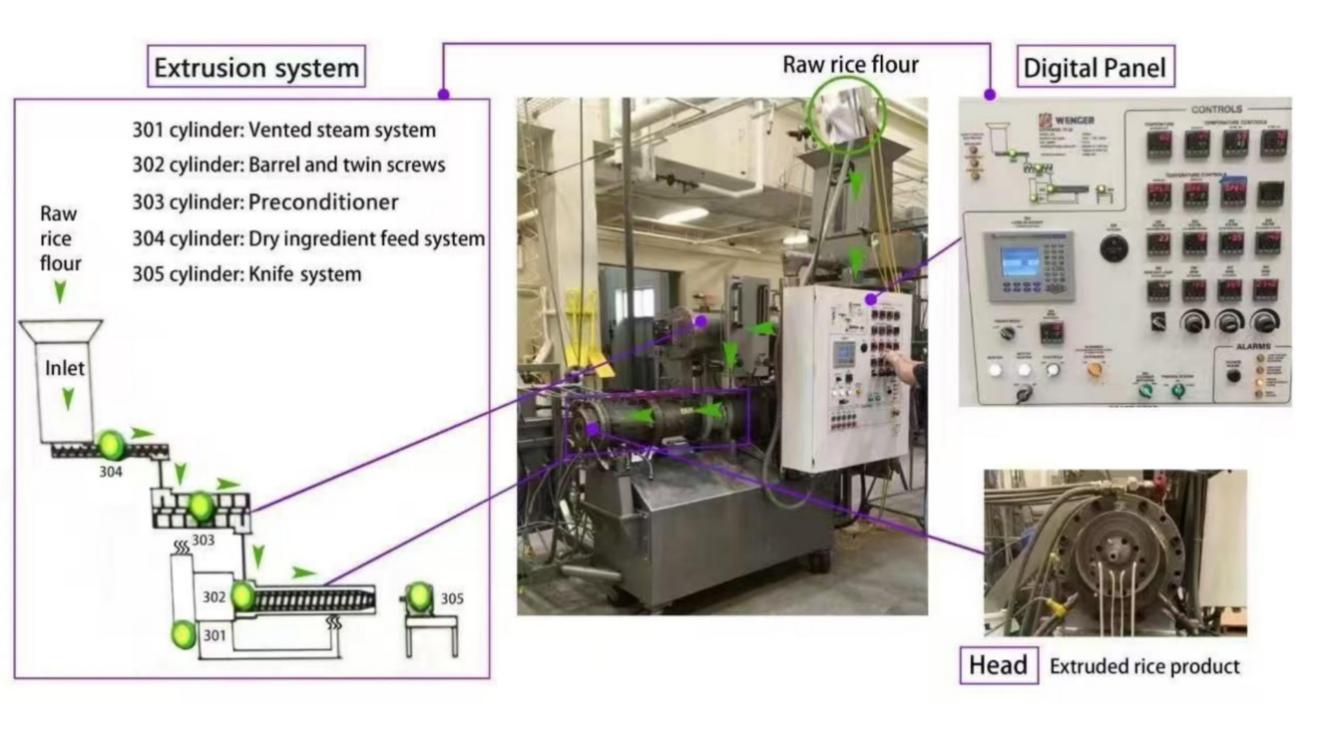
**

**Supplementary Figure 2: Pilot-scale extruder**

**
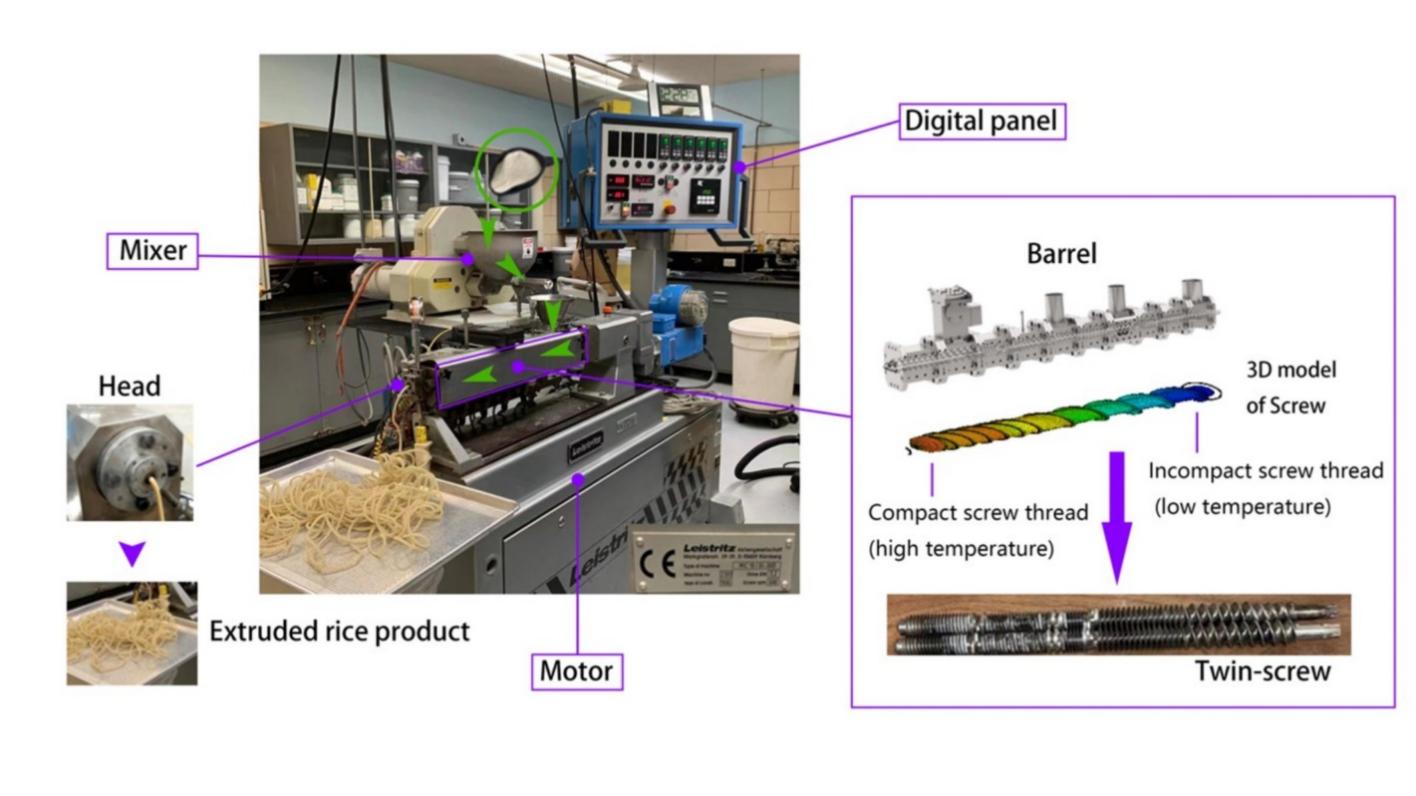
**

**Supplementary Figure 3: Lab-scale extruder**

**
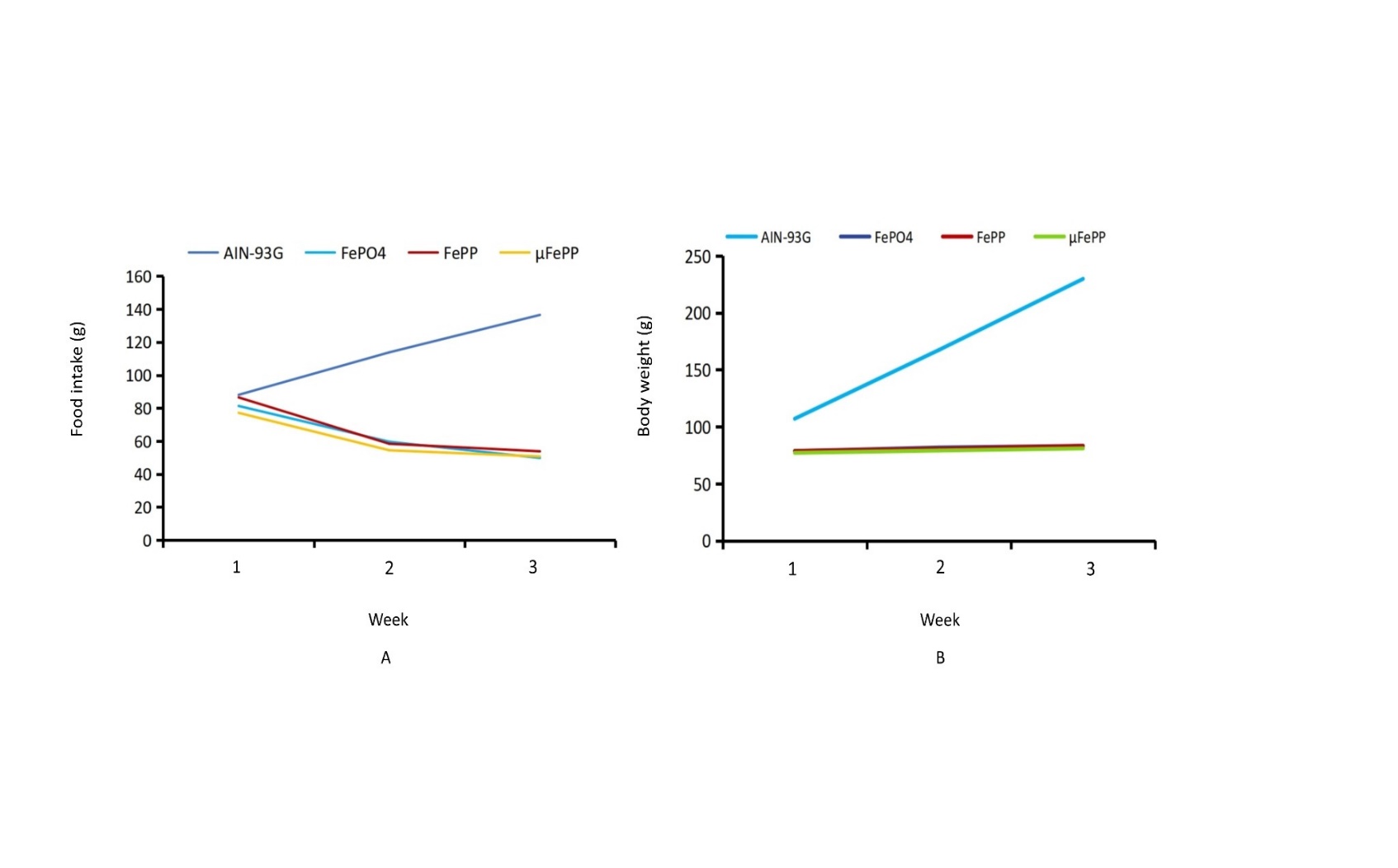
**

**Supplementary Figure 4: Rat Study 1 mean weekly food intake (moisture-adjusted) and mean weekly body weight**

A: All diets reported at 6.6% moisture basis. Total food intake for AIN-93G significantly higher compared to all iron-fortified groups. n=10. B: Total final body weight and total weight gain for AIN-93G significantly higher compared to all iron-fortified groups. n=10.

**
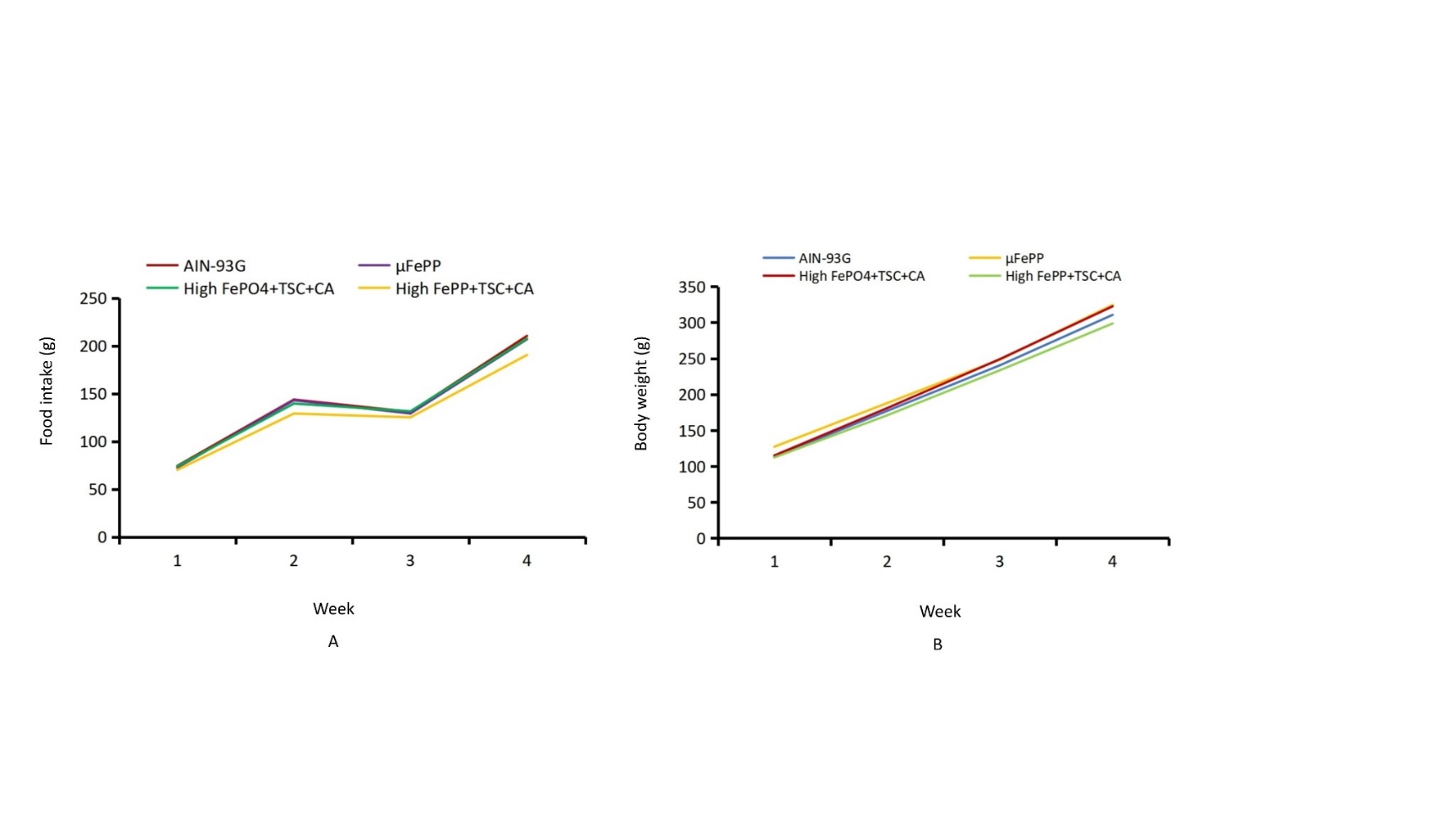
**

**Supplementary Figure 5: Rat Study 2 mean weekly food intake and mean weekly body weight**

A: There was no statistically significant difference in the mean weekly food intakes between the groups. n=10. B: No statistically significant difference was observed in the mean weekly weights between the groups. n=10.
